# Ozone exposure is positively correlated with the occurrence of Kawasaki disease in Chinese children

**DOI:** 10.1038/s41390-025-04116-8

**Published:** 2025-05-13

**Authors:** Huang ShuHan, Huang ZhiMeng, Lin YaXuan, Fang JingXuan, Chen RuiQi, Guo WenXing, Zhang HuiFen, Yang Xiaoqing, JinZhun Wu, Zhong LiLin

**Affiliations:** 1 Department of Neonatology, Women and Children’s Hospital, School of Medicine, Xiamen university, Xiamen, 361003 Fujian China; 2 Department of Pediatrics, Women and Children’s Hospital, School of Medicine, Xiamen University, Xiamen, 361102 Fujian China

## Abstract

**Background:**

We studied whether ozone (O_3_) exposure will affect the occurrence of KD, in Xiamen.

**Methods:**

A time-stratified case-crossover design was conducted to explore the relationship between O_3_ exposure and KD in children. A total of 604 participants from 2017 to 2024 were included. Artificial intelligence technology combined with large data model was used to calculate O_3_ concentration, and O_3_ exposure was assigned to each participant. Poisson generalized additive model was used to calculate the risk effect of O_3_ and KD. Correlation and mediation analysis were used to study the mechanism of KD.

**Results:**

When lag 2 to 6 days, O_3_ exposure will increase the occurrence of KD. On the 4th day of lag, O_3_ led to the highest risk of KD, relative risk(RR) = 1.09(95%CI = 1.008, 1.19). The results of mediation analysis showed that clinical indicators such as white blood cell (WBC), neutrophil (NEUT), and C-reactive protein (CRP) were the main mediators regulating the association between O_3_ and KD.

**Conclusion:**

Our results show that exposure to O_3_ is a potential risk factor for KD in children, and clinical indicators such as WBC, NEUT, PLT and CRP are the main mediators regulating O_3_ and KD.

**Impact:**

- We studied the association between O_3_ exposure and the incidence of KD, and further analyzed the regulatory role of clinical indicators in this association. On the 4th day of lag, O_3_ led to the highest risk of KD, RR = 1.09(95%CI = 1.008,1.19). The relationship between O_3_ exposure and KD is mainly mediated by clinical indicators such as WBC, NEUT, PLT and CRP.
- Our findings explain the association between O3 exposure and the incidence of KD, and further analyze the regulatory role of clinical indicators in the association. It is helpful to provide theoretical support for subsequent research.

## Introduction

KD is a childhood acute febrile systemic vasculitis disease, which occurs in the coronary artery and may lead to coronary dilatation, aneurysm and thrombosis.^1^ KD mainly affects children aged 6 months to 5 years old. It is the main cause of acquired heart disease in children in developed countries and is becoming the main factor of acquired heart disease in developing countries.^2,3^ The global incidence of KD is 100/100,000 per year. In Japan, the incidence rates in 2015 and 2016 were 330.2/100,000 and 309.0/100,000, respectively.^4,5^ However, the incidence rate in China from 2013 to 2017 was 68.8/100,000 to 107.3/100,000, showing an increasing trend.^6^

Related studies have shown that air pollutants such as O_3_, PM_2.5_ and PM_10_ can increase the incidence of diseases such as respiratory system, cardiovascular system, and autoimmune system, and ultimately lead to increased mortality in children.^7,8^ Lin et al. used the generalized additive model combined with the distributed lag nonlinear model to estimate the impact of daily air pollutants on the incidence of KD. The results showed that there was a positive correlation between the incidence of KD and the three main air pollutants, PM_10_, SO_2_ and NO_2_.^2^ A case-control study from 2004 to 2010 in Taiwan, collected 4192 cases of KD, randomized controlled at a ratio of 1:4, the results also showed that CO, NO, NO_2_ were positively correlated with the incidence of KD.^9^

At present, there is still a lack of research on the effect of O_3_ on the pathogenesis of KD.^10^ In this study, we investigated the association between O_3_ exposure and KD incidence and found the main clinical mediators that regulate the association between O_3_ and KD in Xiamen, China for 8 years. It is hoped that it will help readers to understand the possible mechanism and etiology of KD.

## Methods

### Data sources and quality control

**Fig. 1:**
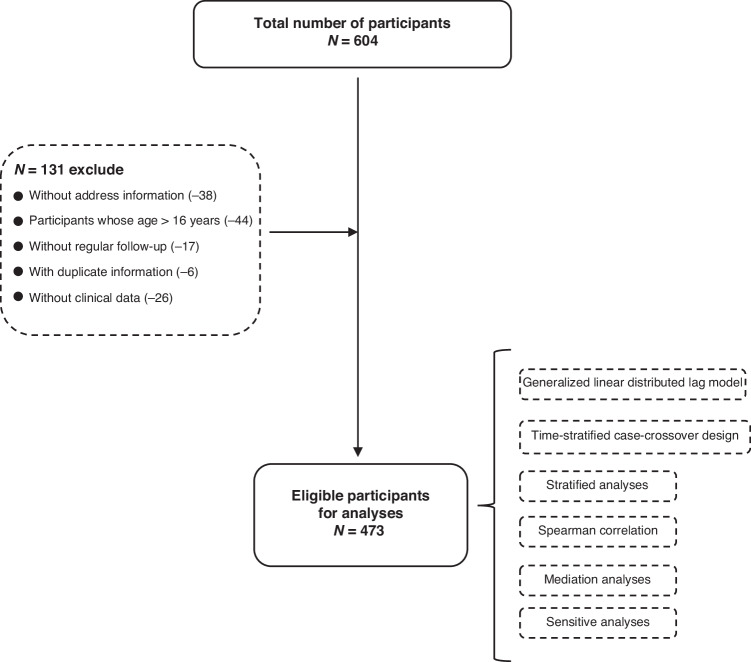
Flow diagram of the study.

**Fig. 2:**
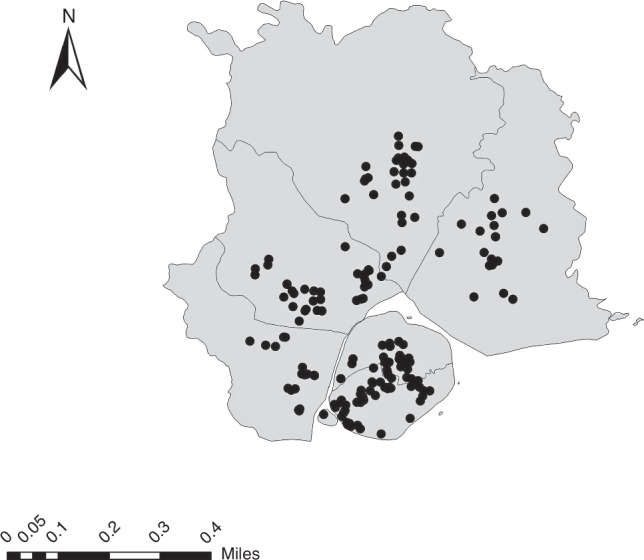
The distribution map of participants in this study.

After excluding the clinical data of atypical and incomplete KD, the clinical data of 604 children with typical KD were collected in Xiamen, China, from January 1,2017 to October 1,2024. We conducted a case-cohort study, followed up pediatric patients regularly, and collected their residential addresses, medical data, demographic status, comorbidities, and other anthropometric and laboratory information (Figs. 1, 2). All participants provided written informed consent at the time of recruitment. The study protocol was approved by the Medical Ethics Committee of Xiamen Maternal and Child Health Hospital (3502Z20224ZD1235).

The current diagnostic criteria for typical KD is persistent fever (≥5days) and at least four of the five clinical symptoms: (1)oral erythema; (2)bilateral conjunctival congestion; (3)rash (maculopapule, diffuse erythroderma, polymorphic erythema); (4)hand and foot erythema/edema/ desquamation; (5)cervical lymphadenopathy.^11^ The disease was coded using the 11th revised edition of the International Classification of Diseases(ICD-11; WHO 2024).^12^

### Exposure assessment

The air pollution dataset comes from China High Air Pollutants (CHAP), a high-resolution near-surface air pollutant dataset in China. The dataset is produced by using artificial intelligence technology, surface solar radiation intensity and air temperature as the main predictors, combined with large data such as ground air pollution monitoring station monitoring data, atmospheric reanalysis and pollutant emission inventory.^13^ Finally, the daily average pollutant exposure concentration at a spatial resolution of 1 km was established. Random 10-fold cross-validation R^2^ value was 0.86.^14^ Based on the residential address of the child, we obtain the geographical coordinates, and use Arcmap10.3 software to perform spatial location matching to calculate the pollutant concentration of each participant’s residence, and obtain the corresponding spatial location pollutant value.

### Study design and statistical analysis

The association between air pollutants and KD was studied using a time-stratified case-crossover design. This research design can control confounding factors and risks that change over time in the short term.^15,16^ The case group was used as its own control group to avoid the deviation of exposure on different dates.^17^ It can also control all known and unknown confounding factors, such as age, weight, socio-economic factors and genetic predisposition. Time series methods have been widely used to study the impact of the environment on adverse health outcomes.^2,18^

Poisson generalized additive model was used to analyze the influence of daily weather conditions on the occurrence of KD. The model can be applied to linear and nonlinear models, and has the advantages of interpretability, flexibility and regularization.^19^ In order to consider the time lag effect of various exposures, the lag time is from the day of fever to 8 days ago, that is, to study the effect of 0–8 days lag of O_3_ on the occurrence of KD.

**Fig. 3:**
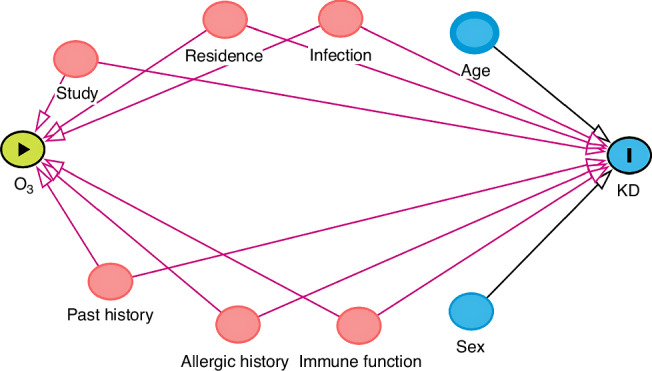
Directed acyclic graph of KD and related risk factors.

In stratified analyses, we considered factors such as sex (male/female), age group (<1year, ≥1year), and school attendance (yes, no) to assess associations in important subgroups. Before considering the multi-pollutant model, we calculated the Spearman correlation between air pollutants. If the correlation coefficient between the two exposed variables is too high (more than 0.7), it is excluded from the analysis.^20^ Directed acyclic graph (DAG) is an intuitive and rigorous tool to identify variables. Controlling variables at the design or analysis stage is sufficient to eliminate confounding factors and some forms of selection bias, and is often used to evaluate causal problems in clinical and epidemiological studies.^21,22^ After referring to the relevant literature of previous studies, we used DAG to evaluate the confounding factors, and considered the following covariates into the final model: age, sex, past history, comorbidities and so on^23–25^ (Fig. 3). In order to determine the causal mediating effect of clinical indicators, we conducted mediating analysis to study the mechanism of KD, and calculated the average direct effect (ADE), the average causal mediating effect (ACME) value and the total effect value (TE).^26^

### Sensitivity analyses

Firstly, the crude model only included the association between O_3_ exposure concentration and KD in children. Model 1 adjusted for age, sex, medical history, blood routine, and other factors based on the crude model; in Model 2, considering the deterioration of air quality due to population migration and extensive firecrackers during the Lunar New Year, we excluded the data of 40 days before and after New Year’s Eve and re-estimated the O_3_ concentration.^27^ Model 3 Because other air pollutants, such as PM2.5, PM10 and temperature, may also affect the occurrence of KD, we adjusted it into the multi-pollutant model.^28,29^ In Model 4, considering that microbial infection may also lead to KD, we excluded participants with infection-induced disease and reanalyzed the association between O_3_ and KD.^30^

Two-tailed probability *P* < 0.05 was considered statistically significant. All analyses were performed using R software version 4.0.5 with software packages “dlnm”, “splines”, “tsModel”, “forestploter” and so on.

## Results

### Characteristics of participants

A total of 604 children with KD were included in this study. 131 subjects were excluded according to the following criteria: (1)Lack of specific address information (*n* = 38); (2)Participants were older than 16 years(*n* = 44); (3)Patients who were lost to follow-up (n = 17); (4)repeated participants (*n* = 6); (5)Lack of clinical data (*n* = 26) (Fig. 1). As a result, a total of 473 subjects were available for analysis. Table 1 shows the descriptive characteristics of the participants in the study, with an average age of 1 year and 2 months. The maximum number of children aged 1 to 3 years old was 313(66.11%), of which male children were more than female children, and male children were 289(61.09%). The number of children attending school is low, at 101(21.34%). The median O_3_ exposure concentration on the first day was 101.55 (81.93,178.5) μg/m^3^.

**Table 1:** Descriptive characteristics of the participants.

	*N*=473
Age	
<1 (y)	59 (12.55%)
1—3 (y)	313 (66.11%)
≥4	101 (21.34%)
Sex	
Male	289 (61.09%)
Female	184 (38.91%)
Weight	11.87 (8.24,14.37)
Study	
Yes	101 (21.34%)
No	372 (78.66%)
Seasons	
Spring	99 (20.92%)
Summer	139 (29.29%)
Autumn	135 (28.45%)
Winter	101 (21.34%)
Habitat	
City	156 (33.05%)
Country	317 (66.95%)
Full term	
Yes	420 (88.7%)
No	53 (11.3%)
Past history	
Yes	150 (31.8%)
No	323 (68.2%)
Infection	
Yes	158 (33.47%)
No	315 (66.53%)
Allergic history	
Yes	46 (9.62%)
No	427 (90.38%)
IgA (g/L)	0.98 (0.36,5.03)
IgG (g/L)	8.12 (5.51,23.4)
IgM (g/L)	1 (0.75,2.22)
C3 (g/L)	1.41 (1.24,2.07)
C4 (g/L)	0.33 (0.27,0.64)
Total T lymphocytes (%)	57.53 (49.33,81.87)
Th cell (%)	32.04 (26.13,53.56)
Tc/Ts cell (%)	21.01 (16.86,50.35)
NK cell (%)	10.52 (6.26,28.73)
B cell (%)	28.99 (22.15,62.97)
Th/Ts cell	1.83 (1.2,4.99)
WBC (×10^9^/L)	14.2 (10.12,32.36)
NEUT (×10^9^/L)	9.4 (5.9,26.39)
LY (×10^9^/L)	3.5 (1.95,9.3)
HGB (g/L)	109.05 (103,131)
PLT (×10^9^/L)	381.08 (293,853)
CRP (mg/L)	76.09 (36.42,252.53)
PT (sec)	14.46 (13,82)
APTT (sec)	34.5 (31.28,47.6)
TT (sec)	13.68 (12.5,37.4)
FIB (ug/ml)	4.94 (4.43,8.88)
DD (ng/mL)	623.07 (310,2662)
LDH (U/L)	292.31 (233,1046)
ALT (U/L)	88.75 (17.28,815.6)
AST (U/L)	84.25 (26.68,1052.9)
1 d ozone exposure (μg/m3)	101.55 (81.93,178.5)
3 d ozone exposure (μg/m3)	100.97 (71.6,171.4)
7 d ozone exposure (μg/m3)	105.3 (86.2,181)

*IgA* Immunoglobulin a, *IgG* Immunoglobulin G, *IgM* Immunoglobulin M, *C3* Complement 3; *C4* Complement 4, *Th cell* t helper cells, *Tc/Ts cell* cytotoxic t lymphocyte/suppressor T cell, *NK cell* natural killer cell, *B cell* bonemarrow derived cell, *Th/Ts cell* t helper cells/suppressor T cell, *WBC* White blood cell, *NEUT* Neutrophils, *LY* lymphocyte, *HGB* Hemoglobin, *PLT* platelet, *CRP* C-reactive protein, *PT* prothrombin time, *APTT* activated partial thromboplastin time, *TT* thrombin time, *FIB* fibrinogen, *DD*d-dimer,* LDH* lactic dehydrogenase, *ALT* alanine aminotransferase, *AST* aspartate aminotransferase.

### Correlation analysis of air pollutants and meteorological factors

**Fig. 4:**
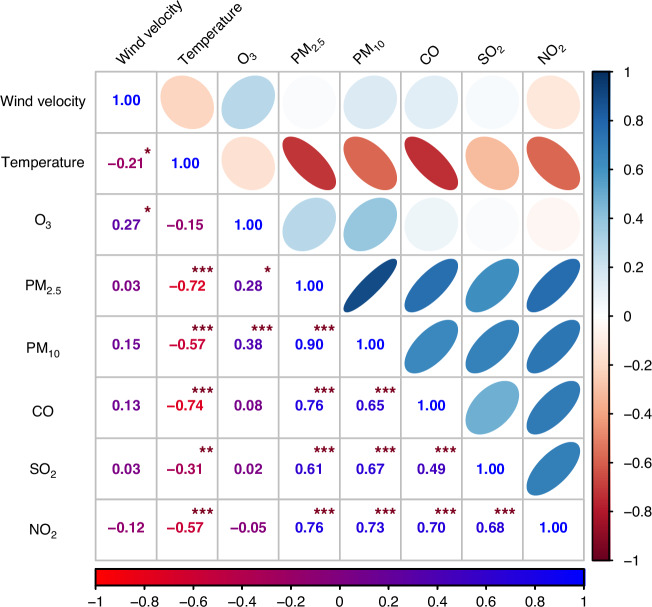
Correlation analysis of air pollutants and meteorological factors.

The correlation between pollutants and meteorological factors was analyzed. The results showed that there was a negative correlation between temperature and wind speed (*P* < 0.05), and the correlation coefficient was −0.21. O_3_ was positively correlated with wind speed (*P* < 0.05), and the correlation coefficient was 0.28. There was a positive correlation between PM_2.5_ and O_3_ (*P* < 0.01), and the correlation coefficient was −0.72. Temperature was negatively correlated with SO_2_, PM_10_, CO and NO_2_ (*P* < 0.05) (Fig. 4).

### Stratified analysis of KD under O_3_ exposure

**Fig. 5:**
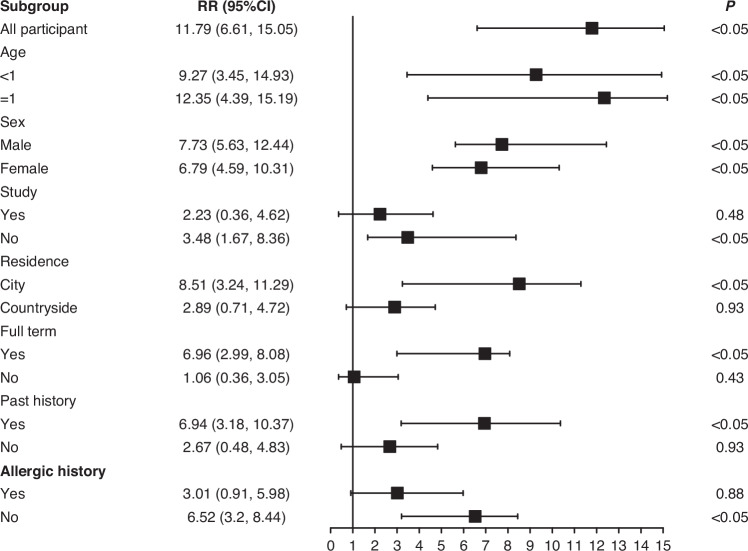
Stratified analysis of KD under O_3_ exposure. RR: relative risk.

Stratified analysis of children ‘s sex, age, past medical history and other factors showed that sex, age and past medical history were risk factors. The age of children was associated with the risk of KD, RR was 9.27 and 12.35, 95%CI = (3.45, 14.93), (4.39,15.19). There was no correlation between the occurrence of KD and no previous history and living in rural areas (Fig. 5).

### Effect of O_3_ exposure on KD and exposure window

**Fig. 6:**
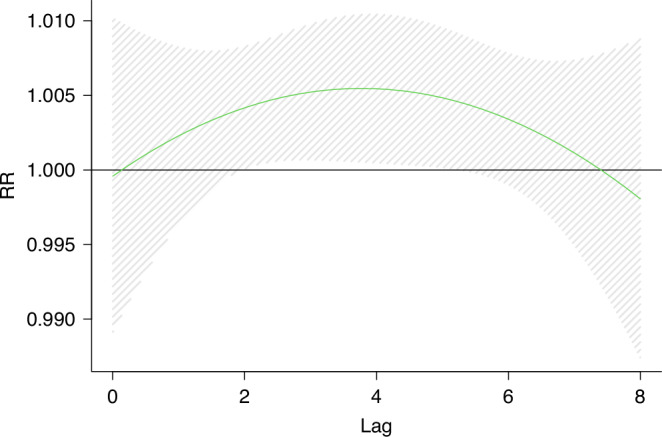
Effect of O_3_ exposure on KD and exposure window(Crude model). The figure shows a correlation between short-term O_3_ exposure and KD. The dashed lines represent a 95% confidence interval. The crude model only included the association between O_3_ exposure concentration and KD in children.

The distribution lag effect model was used to analyze the relationship between O_3_ and KD. Without adjusting the relevant factors, the results showed that the occurrence of KD was significantly correlated with O_3_ from lag 2 days to lag 5 days. On the 2th day of lag, exposure to O_3_ increased the risk of KD by 7.1%(RR = 1.07,95%CI = (1.001,1.15)). On the 4th day of lag, O_3_ led to the highest risk of KD, RR = 1.09(95%CI = 1.008,1.19) (Fig. 6).

### Clinical indicators and KD mediation analysis

The mediating role of related clinical indicators, such as WBC, NEUT and PLT, between O_3_ exposure and KD was evaluated. The results showed that lymphocyte (LY), hemoglobin (HB) and fibrinogen (FIB) had direct mediating effects. Clinical indicators such as WBC, NEUT, PLT, and alanine aminotransferase (ALT) have significant direct and indirect mediating effects. In addition, these clinical indicators are considered to be risk factors for KD (Table 2).

**Table 2:** Clinical indicators and KD mediation analysis.

	ACME	*P*	ADE	*P*	Total Effect	*P*	Proportion Mediated	*P*
IgA	0.00018	0.55	0.001662	0.29	−0.00148	0.32	−0.12161	0.67
IgG	0.000125	0.67	−0.00161	0.29	−0.00148	0.32	−0.08407	0.74
IgM	0.000019	0.46	0.000772	0.66	0.000791	0.65	0.024067	0.72
C3	0.000658	0.37	−0.00214	0.11	−0.00148	0.3	−0.4438	0.64
C4	0.0000245	0.94	−0.00151	0.3	−0.00148	0.35	−0.0166	0.91
Total T lymphocytes	0.000019	0.46	0.000772	0.66	0.000791	0.65	0.024067	0.72
Th cell	−0.000436	0.32	−0.00169	0.32	−0.00212	0.21	0.205391	0.45
Tc/Ts cell	0.000207	0.67	−0.00233	0.12	−0.00212	0.2	−0.09763	0.81
B cell	0.0000241	0.47	−0.00215	0.21	−0.00212	0.21	−0.0114	0.64
Th/Ts cell	0.000019	0.46	0.000772	0.66	0.000791	0.65	0.024067	0.72
WBC	**0.00171**	**<0.05**	**0.00546**	**<0.05**	**0.00717**	**<0.05**	**0.23852**	**<0.05**
NEUT	**0.00217**	**<0.05**	**0.00488**	**<0.05**	**0.00705**	**<0.05**	**0.308**	**<0.05**
LY	−0.0000087	0.92	**0.0071**	**<0.05**	**0.00709**	**<0.05**	−0.00123	0.84
HGB	0.0000104	0.94	**0.00699**	**<0.05**	**0.007**	**<0.05**	0.00148	0.94
PLT	**0.000376**	**<0.05**	**0.00662**	**<0.05**	**0.007**	**<0.05**	**0.0537**	**<0.05**
CRP	**0.000354**	**<0.05**	**0.000578**	**<0.05**	**0.002576**	**<0.05**	**0.000846**	**<0.05**
PT	**0.00154**	**<0.05**	**0.00525**	**<0.05**	**0.00679**	**<0.05**	**0.2269**	**<0.05**
APTT	**0.000493**	**<0.05**	**0.00644**	**<0.05**	**0.00693**	**<0.05**	**0.0712**	**<0.05**
TT	**0.002046**	**<0.05**	**0.004821**	**<0.05**	**0.006867**	**<0.05**	**0.297935**	**<0.05**
FIB	**0.004321**	**<0.05**	**0.002184**	**<0.05**	**0.006505**	**<0.05**	**0.664317**	**<0.05**
DD	0.000638	0.072	**0.00609**	**<0.05**	**0.00672**	**<0.05**	0.0948	0.072
LDH	0.0000194	0.78	**0.00701**	**<0.05**	**0.00703**	**<0.05**	0.00276	0.78
ALT	**0.000668**	**<0.05**	**0.006549**	**<0.05**	**0.007217**	**<0.05**	**0.092505**	**<0.05**
AST	**0.000236**	**<0.05**	**0.00702**	**<0.05**	**0.00725**	**<0.05**	**0.0326**	**<0.05**

*IgA* Immunoglobulin a,* IgG* Immunoglobulin G*,* *IgM* Immunoglobulin M, *C3* Complement 3, *C4* Complement 4, *Th cell* t helper cells, *Tc/Ts* *cell* cytotoxic t lymphocyte/suppressor T cell, *B cell* bonemarrow derived cell, *Th/Ts cell* t helper cells/suppressor T cell, *WBC* White blood cell, *NEUT* Neutrophils, *LY* lymphocyte, *HGB* Hemoglobin, *PLT* platelet, *CRP* C-reactive protein,* PT* prothrombin time, *APTT* activated partial thromboplastin time, *TT* thrombin time, *FIB* fibrinogen, *DD* d-dimer, *LDH* lactic dehydrogenase, *ALT* alanine aminotransferase, *AST* aspartate aminotransferase, *ACME* Average Causal Mediation Effect, *ADE* Average Direct Effect.

Bold values represent statistical significance *p* < 0.05.

### Sensitivity analysis

**Fig. 7:**
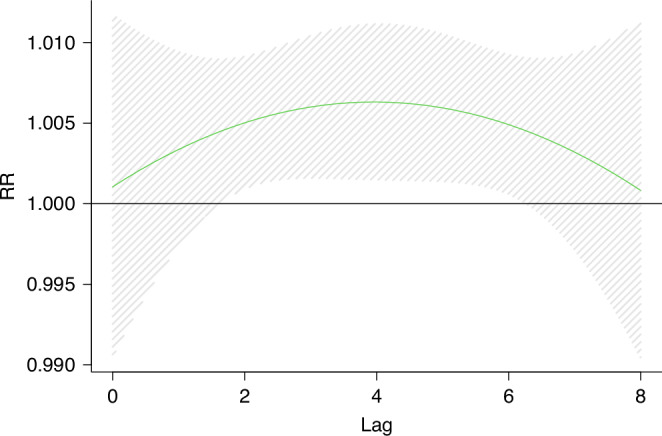
Effect of O_3_ exposure on KD and exposure window(model1). The figure shows a correlation between short-term O_3_ exposure and KD. The dashed lines represent a 95% confidence interval. Model 1 adjusted for age, sex, medical history, blood routine, and other factors based on the crude model.

**Fig. 8:**
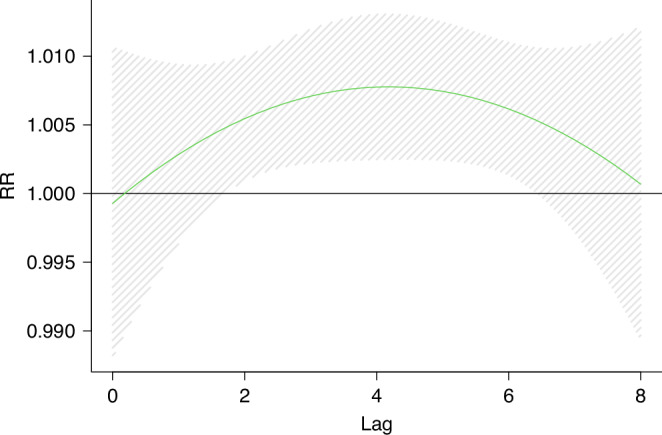
Effect of O_3_ exposure on KD and exposure window(model2). The figure shows a correlation between short-term O_3_ exposure and KD. The dashed lines represent a 95% confidence interval. In model 2, we excluded the data of 40 days before and after New Year’s Eve and re-estimated the O_3_ concentration.

**Fig. 9:**
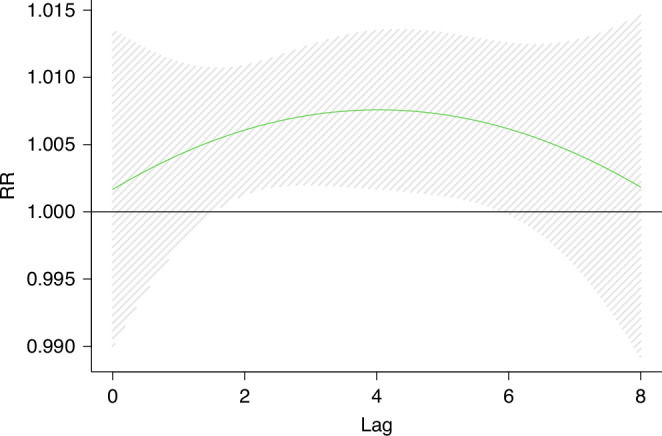
Effect of O_3_ exposure on KD and exposure window(model3). The figure shows a correlation between short-term O_3_ exposure and KD. The dashed lines represent a 95% confidence interval. In model 3, we included the multi-pollutant model for adjustment.

**Fig. 10:**
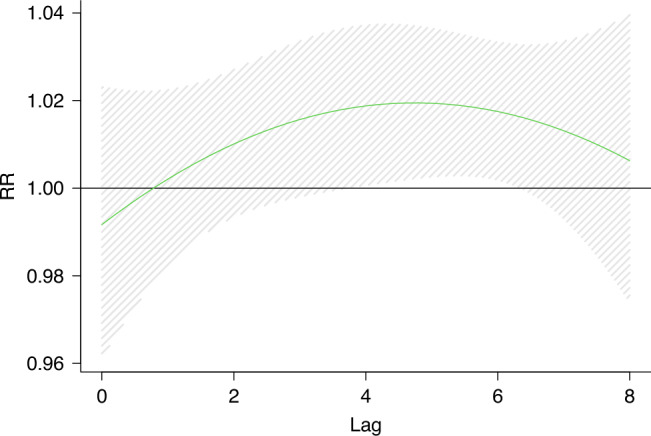
Effect of O_3_ exposure on KD and exposure window(model4). The figure shows a correlation between short-term O_3_ exposure and KD. The dashed lines represent a 95% confidence interval. In model 4, we excluded participants with infection-induced disease.

In Model 1, the age, sex, allergy history and other factors of the children were adjusted as covariates, and the results remained stable. In model 2, due to the high daily average value of O_3_ during the Chinese New Year, we excluded O_3_ exposure for 40 days before and after Lunar New Year ‘s Eve, and reassessed the association between O_3_ and KD. The results did not change substantially.^31^ Model 3, a variety of pollutant models showed that the effect of O_3_ exposure on KD was enhanced, but the change was not significant. Model 4, excluding the participants caused by infection, the model remains stable. All sensitivity analyses confirm the robustness of our main conclusions (Table 3 and Figs. 6–10).

**Table 3:** Sensitivity analysis of different models.

	lag day	1 day	2 day	3 day	4 day	5 day	6 day	7 day
Crude Model	RR (95%CI)	0.96 (0.94,1.15)	**1.07 (1.001,1.15)**	**1.09 (1.01,1.18)**	**1.09 (1.01,1.19)**	**1.08 (1.002,1.18)**	0.98 (0.94,1.14)	0.98 (0.92,1.13)
	*P* value	0.077	<0.05	<0.05	<0.05	<0.05	0.38	0.091
Model 1	RR (95%CI)	0.96 (0.93,1.17)	**1.09 (1.02,1.17)**	**1.10 (1.03,1.19)**	**1.11 (1.03,1.21)**	**1.10 (1.02,1.19)**	**1.08 (1.01,1.17)**	0.94 (0.90,1.17)
	P value	0.26	<0.05	<0.05	<0.05	<0.05	<0.05	
Model 2	RR (95%CI)	0.96 (0.93,1.16)	0.94 (0.97,1.15)	0.92 (0.97,1.19)	0.99 (0.90,1.22)	**1.12 (1.03,1.24)**	**1.15 (1.04,1.30)**	0.97 (0.82,1.48)
	*P* value	0.084	0.33	0.067	0.58	<0.05	<0.05	0.65
Model 3	RR (95%CI)	0.96 (0.93,1.21)	**1.10 (1.02,1.20)**	**1.12 (1.03,1.23)**	**1.13 (1.03,.26)**	**1.12 (1.02,1.25)**	0.99 (0.89,1.24)	0.93 (0.84,1.24)
	*P* value	0.24	<0.05	<0.05	<0.05	<0.05	0.057	0.24
Model 4	RR (95%CI)	0.96 (0.73,1.46)	0.89 (0.83,1.58)	0.96 (0.73,1.76)	**1.32 (1.01,1.87)**	**1.33 (1.04,1.85)**	**1.30 (1.03,1.75)**	0.89 (0.78,1.76)
	*P* value	0.89	0.47	0.92	<0.05	<0.05	<0.05	0.65

*RR* relative risk;Crude model only included the association between O3 exposure concentration and KD in children; Model 1 adjusted for age, sex, medical history, blood routine, and other factors based on the crude model; Model 2, we excluded the data of 40 days before and after New Year’s Eve and re-estimated the O3 concentration; Model 3 we adjusted it into the multi-pollutant model; Model 4 we excluded participants with infection-induced disease and reanalyzed the association between O3 and KD.

Bold values represent statistical significance *p* < 0.05.

## Discussion

Environmental temperature, ultraviolet radiation and humidity are the key factors affecting O_3_ concentration. The increase of O_3_ concentration will lead to an increase in the incidence of chronic diseases.^32,33^ A six-year study has shown that short-term O_3_ exposure increases the risk of death at different temperature levels.^34^ Related studies have shown that O_3_ may be a risk factor, which can induce oxidative stress, cause acute inflammatory response, and lead to systemic autoimmune response.^35,36^ In the acute phase of KD, the excessive production of reactive oxygen species in the body due to related incentives will destroy the balance between oxidation and anti-oxidation (reduction) reactions, thereby triggering a vicious cycle of inflammatory reactions and reactive oxygen metabolites, forming diffuse vasculitis, leading to coronary artery damage.^37^ A study counted the degree of coronary artery dilatation in 282 children with KD in Australia. The results showed that only 47(16.7%) had coronary artery dilatation and 19(6.8%) had coronary artery aneurysm.^38^Preschool children, urban residents, and increased outdoor activities may be high risk factors, which is consistent with our stratified analysis.^39,40^

Studies have shown that cytokine storms such as IL-6, IL-10, IFN-α, IFN-γ, IL-1β, and IL-1 activation are associated with the severity of autoimmune diseases.^41–43^ KD activates the innate immune response and increases the number of T helper cells.^44^ However, related animal experiments have shown that mice lacking T cells still have moderate to severe cardiac inflammation, suggesting that T cells may not be necessary in the development of KD vasculitis.^45^ The autopsy of several KD patients in the United States showed that the amount of IgA produced in the tissue and coronary artery wall of KD patients was higher than that of other patients. However, our research results have no relevant support.^46^ Regarding the mechanism of coronary artery disease in KD, platelet-derived growth factor is released into the vascular wall when endothelial cells are damaged, which may lead to changes in coronary inflammation.^47^ In the early stage of KD, NEUT can express inducible nitric oxide synthase(iNOs), which synthesizes vasodilator factors and leads to early coronary endothelial dysfunction in children with KD.^48,49^ The results of this study show that PLT and NEUT have significant direct and indirect mediating effects.

Another population-based cohort study in Taiwan found that the incidence of KD in children increased after infection with enterovirus.^50^ A clinical study in China showed that the increase of Fusobacterium, Shigella and Streptococcus may increase the incidence of KD. Bifidobacterium, Lactobacillus and Roseburia may help to alleviate the incidence of KD.^51^ The results of sensitivity analysis in this paper also showed that infection may lead to an increase in the incidence of KD, similar to our findings. Cohort studies in Japan and China showed that SO_2_ was significantly associated with increased KD incidence.^2,30^ However, another study of 3009 KD cases in seven major cities in North America observed that the effect of PM2.5 exposure on KD was not statistically significant.^52^ From 2013 to 2020, a cohort study in eastern China showed that there was a significant correlation between O_3_ exposure and KD incidence at lag 0 days, while lag 1 days and lag 2 days became statistically insignificant.^53^ In our study, after expanding the lag time, the results showed that O_3_ had different degrees of influence on KD in lag 2–5 days, and it was statistically significant.

## Conclusions

In this study, we found that exposure to O_3_ was associated with an increased risk of KD. On the 4^th^ day of lag, O_3_ led to the highest risk of KD, RR = 1.09(95%CI = 1.008,1.19). Further stratified analysis confirmed that a stronger positive correlation was observed in males, living in urban areas, and children with previous medical history. The relationship between O_3_ exposure and KD is mainly mediated by clinical indicators such as WBC, NEUT, PLT and CRP. Our results suggest that controlling industrial air pollutants, especially O_3_ emissions, may help to curb the disease burden of KD.

### Importance and limitations

This study has some advantages. First of all, our professional doctors use relevant scales to diagnose and classify diseases, and regular follow-up questionnaires are conducted by specialized nurses.^54^ Inspection and analysis were carried out by relevant statisticians. Secondly, we used artificial intelligence technology and large data model technology to accurately expose O_3_ to the residential address of each participant, providing the accuracy of the air pollution exposure value of the child.^14^ In the sensitivity analysis, the confounding factors of infectious diseases and pathogenic microorganisms were excluded, and the date of onset was inferred through the chief complaint of the child, which increased the reliability of the results. Finally, this study shows that the relationship between O3 and KD is mainly mediated by WBC, NEUT, PLT and CRP, which is helpful for our further research.

Of course, our current research also has some limitations. First of all, our research is based on the residential address of the participants, but some participants may participate in outdoor sports, which may affect the accuracy of the results.^55^ Secondly, due to differences in geographical location, target population and research design, the validity or universality of our results may be another major issue. Our study did not exclude medical records during the novel coronavirus epidemic. Relevant studies have shown that the novel coronavirus may increase the incidence of KD, which may also lead to deviations in the results.^56,57^ Finally, this epidemiological study cannot answer the molecular mechanism of KD caused by O_3_ exposure, so further toxicological studies are needed to confirm our preliminary findings.

## Data Availability

The datasets generated and analysed during the current study are not publicly available due patient privacy but are available from the corresponding author on reasonable request.
